# ALKBH5 in mouse testicular Sertoli cells regulates *Cdh2* mRNA translation to maintain blood–testis barrier integrity

**DOI:** 10.1186/s11658-022-00404-x

**Published:** 2022-11-22

**Authors:** Zhonglin Cai, Yao Zhang, Lin Yang, Chunhui Ma, Yi Fei, Jing Ding, Wei Song, Wei-Min Tong, Yamei Niu, Hongjun Li

**Affiliations:** 1 Department of Urology, Peking Union Medical College Hospital, Chinese Academy of Medical Sciences & Peking Union Medical College, Beijing, China; 2 Department of Pathology, Institute of Basic Medical Sciences Chinese Academy of Medical Sciences, School of Basic Medicine Peking Union Medical College, Beijing, China; 3 Department of Urology, Shanghai Ninth People’s Hospital, Shanghai Jiaotong University School of Medicine, Shanghai, China; 4 Department of Biochemistry and Molecular Biology, State Key Laboratory of Medical Molecular Biology, Institute of Basic Medical Sciences Chinese Academy of Medical Sciences, School of Basic Medicine Peking Union Medical College, Beijing, China; 5 Molecular Pathology Research Center, Chinese Academy of Medical Sciences and Peking Union Medical College, Beijing, China

**Keywords:** RNA *N*^6^-methyladenosine, *Alkbh5*, Blood–testis barrier, *Cdh2*, Basal endoplasmic specialization

## Abstract

**Background:**

RNA *N*^6^-methyladenosine (m^6^A) is involved in mammalian spermatogenesis. In both germ cells and Leydig cells, ALKBH5 regulates spermatogenesis and androgen synthesis in an m^6^A-dependent manner. However, it is unclear whether ALKBH5 plays a role in testicular Sertoli cells, which constitute the blood–testis barrier (BTB) through cell junctions between adjacent Sertoli cells.

**Methods:**

ALKBH5 expression in the testes of humans and mice was detected by immunohistochemical staining and immunofluorescence staining. BTB integrity was evaluated by BTB assay. m^6^A-seq was performed to screen for BTB-related molecules regulated by ALKBH5. m^6^A immunoprecipitation–quantitative real-time polymerase chain reaction (qPCR), RNA immunoprecipitation–qPCR, western blot, coimmunoprecipitation, and polysome fractionation–qPCR analyses were performed to explore the mechanisms of ALKBH5 in BTB. Transmission electron microscopy was applied to observe the BTB ultrastructure.

**Results:**

ALKBH5 in Sertoli cells is related to the integrity of the BTB. Subsequently, the m^6^A level on *Cdh2* mRNA, encoding a structural protein N-cadherin in the BTB, was found to be regulated by ALKBH5. IGF2BP1/2/3 complexes and YTHDF1 promoted *Cdh2* mRNA translation. In addition, we found that basal endoplasmic specialization, in which N-cadherin is a main structural protein, was severely disordered in the testes of *Alkbh5*-knockout mice.

**Conclusions:**

Our study revealed that ALKBH5 regulates BTB integrity via basal endoplasmic specialization by affecting *Cdh2* mRNA translation.

**Supplementary Information:**

The online version contains supplementary material available at 10.1186/s11658-022-00404-x.

## Introduction

Cumulative studies have indicated that RNA m^6^A is involved in almost every main process in spermatogenesis [1–5]. As reported thus far, spermatogonial stem cell differentiation and maintenance are regulated by METTL3/14 [2], mitosis initiation is regulated by ALKBH5 and YTHDC1, and mitosis maintenance is regulated by METTL3/14. In addition, spermiogenesis is regulated by ALKBH5 [3]. In the process of spermatogenesis, testicular somatic cells, mainly Leydig cells and Sertoli cells, play important roles in the maintenance and regulation of spermatogenesis [6]. Leydig cells regulate spermatogenesis through androgen and estrogen production. Sertoli cells have roles in barrier protection, nutrition, and support for germ cells [7]. ALKBH5 and METTL14 are expressed not only in spermatogenic cells but also in Leydig cells, both of which are jointly involved in the regulation of testosterone synthesis in an m^6^A-dependent manner [8]. However, the role of RNA m^6^A modification in Sertoli cells remains unknown.

Adjacent Sertoli cells near the basal lamina of the seminiferous tubules can form the blood–testis barrier (BTB), which is critical to maintain the unique microenvironment for normal spermatogenesis via cell junctions [9]. Cell junctions are composed mainly of structural proteins, scaffold proteins, and cytoskeletal proteins [10]. The maintenance of normal BTB function depends on the normal expression and function of these proteins [10, 11]. Since ALKBH5 is expressed in mouse testicular Sertoli cells [1], we wanted to determine whether ALKBH5 in Sertoli cells is involved in the regulation of the BTB.

In this study, we mainly explored the relationship between ALKBH5 in Sertoli cells and the integrity of the BTB. Ultimately, we found that ALKBH5 in Sertoli cells is involved in regulating the m^6^A level of *Cdh2* mRNA in basal endoplasmic specializations and regulates the translation of its corresponding protein, N-cadherin, via IGF2BP1/2/3 complexes and YTHDF1 and thus participates in the regulation of BTB integrity.

## Materials and methods

### Cell lines and experimental animals

The TM4 mouse testicular Sertoli cell line (4201MOU-CCTCC00624) was purchased from the National Infrastructure of Cell Line Resource (Beijing, China) and cultured with DMEM/F12 (Gibco, #C11330500) containing 5% horse serum (Solarbio, #S9050) and 2.5% fetal bovine serum (NEWZERUM, #FBS-S500). Two-month-old wild-type C57BL/6 mice were purchased from Vital River. Four-month-old *Alkbh5*-deficient mice were used and genotyped as described previously [12]. All animal experiments and euthanasia were approved and performed in accordance with the guidelines of the Animal Care and Use Committee of IBMS, CAMS.

### Antibodies and primers for RT–qPCR

In this study, all antibodies, including primary and secondary antibodies, are listed in Additional file 1: Table S1. All primers for RT–qPCR in this study are listed as follows: *Gapdh*-forward: 5′-ACAACTTTGGCATTGTGGAA-3′, *Gapdh*-reverse: 5′-GATGCAGGGATGATGTTCTG-3′; *Cdh2-*forward: 5′-CACACCCTGGGGATATTGGG-3′, *Cdh2-*reverse: 5′-GCTGCCCTCGTAGTCAAAGA-3′; *GLuc*-forward: 5′-CGACATTCCTGAGATTCCTGG-3′, *GLuc*-reverse: 5′-TTGAGCAGGTCAGAACACTG-3′; *CLuc*-forward: 5′-GCTTCAACATCACCGTCATTG-3′, *CLuc*-reverse: 5′-CACAGAGGCCAGAGATCATTC-3′.

### Western blotting (WB) analysis

The protein was extracted, and an appropriate amount of denatured protein was used for electrophoresis and electrically transferred to a blotting membrane. After incubating the membrane with 5% blocking solution for 1 h, the primary antibody against each specific protein of interest was incubated with the membrane overnight at 4 °C. Then, the appropriate secondary antibody was incubated with the membrane at room temperature for 1 h or overnight at 4 °C. Finally, the membrane was visualized with an enhanced chemiluminescence reagent (Tanon, #180–501).

### Immunohistochemical staining (IHC) and immunofluorescence staining (IF)

After fixation with 4% paraformaldehyde (PFA) (Sigma, #158127), testis tissue was embedded in paraffin for IHC or optimal cutting temperature compound (OCT) for IF and sliced. For IHC, sections were dewaxed with xylene and rinsed sequentially with 100%, 95%, and 75% ethanol. Then, the sections were heated in citric acid at 95 °C for 10 min for antigen retrieval. Subsequently, endogenous catalase was blocked by treatment with 3% hydrogen peroxide at room temperature for 10 min. Sections were then incubated with primary antibodies (the antibodies used are listed in Additional file 1: Table S1), followed by horseradish-peroxidase-labeled secondary antibodies. Finally, the sections were stained with diaminobenzidine and counterstained with hematoxylin. For IF, sections were blocked with 5% bovine serum albumin (BSA) (Jingke Hongda, 218054991) with 0.5% Triton X-100 (Sigma, #282103) for 1 h, followed by incubation with primary antibody overnight at 4 °C. The tissue was then washed with 1× PBS and incubated with the secondary antibody at room temperature for 1 h. The sample was then counterstained with DAPI (Sigma, #F6057). Multilabel IF was performed by using an immunofluorescence kit from Panovue (#10236100050). Briefly, after the first incubation with primary and secondary antibodies, the sections were washed and blocked with 5% BSA with 0.5% Triton X-100 again. Another primary antibody at a certain dilution ratio was incubated overnight at 4 °C. After 1× PBS washing, the sections were incubated with secondary antibody for 1 h at room temperature, and then DAPI was used to stain the nuclei.

### Blood–testis barrier permeability assay (BTB assay)

A BTB assay was carried out according to protocols published in the literature [13]. Briefly, the testes of anesthetized mice were exposed, and 10 mg/ml EZ-Link Sulfo-NHS-LC-Biotin (Thermo, #21335) was administered to the testes by interstitial injection. After 30 min, the mice were euthanized, and then the testes were removed, fixed with 4% PFA, and embedded in OCT. After the testes were sliced at a thickness of 5 µm and blocked with 5% BSA, Fluor-568-conjugated streptavidin (Invitrogen, #S11226) was applied to the incubated sections and incubated at room temperature for 30 min, and then DAPI was added to stain the sections.

### Small interfering RNA (siRNA) transfection

siRNA transfection was performed using an RFect transfection kit (Changzhou Bio-generating Biotechnology, #11012). Briefly, after cells were seeded in a dish and cultured for 24 h, an appropriate volume of RFect was mixed with serum-free medium and incubated at room temperature for 5 min. An appropriate amount of siRNA was mixed with serum-free medium and incubated with the diluted RFect at room temperature for 20 min and then added to the culture dish. The transfection efficiency was evaluated after 24–72 h. The siRNA sequences used in this study were as follows: scramble: 5′-GGCUCUAGAAAAGCCUAUGC-3′, si*Alkbh5*-1: 5′-ACAAGUACUUCUUCGGCGA-3′, si*Alkbh5*-2: 5′- GCUGCAAGUUCCAGUUCAA-3′, si*Igf2bp1*-1: 5′-CCAUCCGAAACAUCACAAA-3′, si*Igf2bp1*-2: 5’-GAGCAAGUGAACACUGAAA-3′, si*Igf2bp3*-1: 5′-CCAAGCAGAAACCCUGUGA-3′, si*Igf2bp3*-2: 5′-GUGAACACGGAUUCGGAAA-3′, siIgf2bp2-1: 5′-GAAUCCAGAUUCGGAACAU-3′, siIgf2bp2-2: 5′-CGGUUACUCAAGCGAACAA-3′, si*Ythdf1*-1: 5′- GCACUGACUGGUGUCCUUU-3′, si*Ythdf1*-2: 5′- GGAAAUGCCCAACCUACUU-3′. Silencing of *Alkbh5* was performed by a mixture of si*Alkbh5*-1 and si*Alkbh5*-2.

### Transmission electron microscopy

The tissue was fixed with an appropriate amount of 2.5% glutaraldehyde and incubated overnight at 4 °C. Ultrathin sections were stained with 1% uranyl acetate and 0.1% lead citrate and then observed under a transmission electron microscope.

### m^6^A-immunoprecipitation (IP) sequencing (m^6^A-seq) and m^6^A IP-qPCR and

Total RNA was extracted according to the instructions of the TRIzol reagent manufacturer (Invitrogen, #15596026). Total RNA (20 µg) was fragmented into ~ 100–200 nt fragments using RNA fragmentation agents (Thermo Fisher, #AM8740). Fragmented RNA was incubated with anti-m^6^A antibody at 4 °C for 4 h, Dynabeads Protein A was added to mix for immunoprecipitation (Thermo Fisher, #10002D) at 4 °C for 2 h. Beads were washed five times with IPP buffer (150 mM NaCl, 0.1% NP-40, 10 mM Tris–HCl, pH 7.4), and immunoprecipitated RNA was recovered through elution with m^6^A nucleotide followed by ethanol precipitation. When m^6^A-seq was performed, precipitated RNA was used with SMARTer Stranded Total RNA-Seq Kit v2—Pico Input Mammalian (Takara, #634414) for library construction, followed by second-generation sequencing on an Illumina X Ten platform. Two sets of biological duplicate samples were subjected to m^6^A-seq. When qPCR was performed, the precipitated RNA was reverse-transcribed into cDNA using ReverTra Ace qPCR RT master mix with gDNA remover (Toyobo, #fsq-301), and qPCR was performed using Thunderbird SYBR qPCR mix (Toyobo, #qps-201).

### Bioinformatic analysis of m^6^A-seq data

Differential methylation analysis and alternative splicing analysis were performed in a similar way as described in our previous study [14]. The clean reads of each sample were mapped against the mouse genome (mm10). Only uniquely mapped reads were subjected to the subsequent analyses. The gene expression levels were evaluated using the reads per kilobase of transcript per million mapped reads values. The genes that were differentially expressed (|log_2_FC|> 0.58, *P* < 0.05) between the scramble and si*Alkbh5* samples were identified using the DESeq2 R package [15]. The exomePeak R package [16] was used to identify m^6^A peaks in each sample, and HOMER software was used to determine the conserved motifs within these regions. Furthermore, we divided the 3′ untranslated region (UTR), coding sequence region (CDS), and 5′UTR regions of the longest transcript of each gene into 100 equally sized bins separately to characterize the distribution patterns of m^6^A peaks. The percentage of m^6^A peaks in each bin was calculated to represent the occupancy of m^6^A along with the transcripts. Differentially methylated regions between the scramble and si*Alkbh5* samples were further identified using exomePeak software by taking the cutoff of |log_2_FC|> 0.58 and false discovery rate (FDR) < 0.05. Gene Ontology (GO) analysis of the differentially expressed or modified RNAs was conducted on the basis of the DAVID online annotation database. [17, 18] Visualization of the enriched GO terms was implemented using the ggplot2 R package. The RNA-seq data obtained from the scramble and si*Alkbh5* samples were utilized to detect alternative splicing (AS) events using the rMATS tool [19]. The inclusion levels of each event were quantified by the percent spliced in, which was calculated according to the inclusion junction counts and skipping junction counts in each splicing event. The AS events with an FDR < 0.05.

### RNA immunoprecipitation (RIP)–qPCR

RIP was performed as previously reported [14]. Briefly, the cells were collected and lysed with undenatured lysate on ice for 30 min. After centrifugation, the supernatant was collected, and the protein was quantified with a bicinchoninic assay kit (Genstar, #RE162-05) following the manufacturer’s instructions. An appropriate amount of supernatant was mixed with primary antibody and incubated at 4 °C for 6 h. Then, an appropriate amount of BSA-blocked protein A/G magnetic beads (Thermo Scientific, #26162) was added in previous mixture including primary antibody and supernatant and incubated at 4 °C overnight. After being washed twice with low-salt Tris buffer and high-salt buffer, the magnetic beads were resuspended in lysate buffer, and an appropriate amount of the sample was used for WB to verify the IP efficiencies. The remaining magnetic beads were collected, and the proteins were eluted with protein K buffer at 55 °C for 30 min. The precipitated RNA was collected with a RNEasy MinElute cleanup kit (Qiagen, #74204). The precipitated RNA was reverse transcribed into cDNA using ReverTra Ace qPCR RT master mix with gDNA remover (Toyobo, #fsq-301), and qPCR was performed with Thunderbird SYBR qPCR mix (Toyobo, #qps-201). Related mRNA level was calculated, as follows: 2^(Ct_IP_ − Ct_IgG_), and then Student’s *t*-test was used to analyze statistical differences between the groups.

### Coimmunoprecipitation

Coimmunoprecipitation was performed using an immunoprecipitation kit (Beyotime, P2179S) according to the manufacturer’s instructions. Briefly, the cells were lysed and then incubated with the primary antibody at 4 °C overnight; then, Protein A + G beads were added to the cell lysate and primary antibody mixture for 1 h at room temperature, and the beads were washed with 1× TBS. After that, SDS–PAGE was applied to elute protein from the beads.

### Polysome fractionation and RT–qPCR analysis

After treatment with cycloheximide D (Sigma, #c4859) at 37 °C for 10 min, approximately 2 × 10^7^ cells were taken from each sample and lysed. The cell lysate was added to a 20–50% sucrose gradient and centrifuged at 4 °C and 38,000 rpm for 3 h. The supernatant was fractionated into 38 tubes through a gradient fractionator. The efficiency of fractionation was determined through the detection of RPS6 and RPL11 expression in different pipe fractions by WB. RT–qPCR was performed to detect the abundance of the target gene in each fraction.

### Construction of a mouse model with *Alkbh5* knockdown specifically in Sertoli cells

Two-month-old C57BL/6 male mice were used to establish an in vivo *Alkbh5* knockdown model by infection with AAV8-sh*Alkbh5*-GFP (sh*Alkbh5*: ACAAGTACTTCTTCGGCGA) or AAV8-scramble-GFP generated by WZ Biosciences Inc. After anesthesia, the abdominal cavity was opened to expose the testis, and the output tubule was found under a postural microscope. A certain volume of the aforementioned viruses was taken up with a glass pulling needle and injected into the seminiferous tubule. The testis was placed into the scrotum, and the abdominal incision was sutured. The mice were housed in a specific-pathogen-free environment for more than 1 month before used for subsequent analysis.

### Statistical analysis

Data are expressed as the mean ± standard deviation (SD). GraphPad Prism 7 (GraphPad Software Inc., San Diego, CA, USA) and SPSS 22.0 (SPSS Inc., Chicago, IL, USA) were used to conduct statistical analyses. Differences between the groups were analyzed using Student’s *t*-test. *P* < 0.05 was considered statistically significant.

## Results

### ALKBH5 in testicular Sertoli cells is involved in the regulation of BTB integrity

**Fig. 1:**
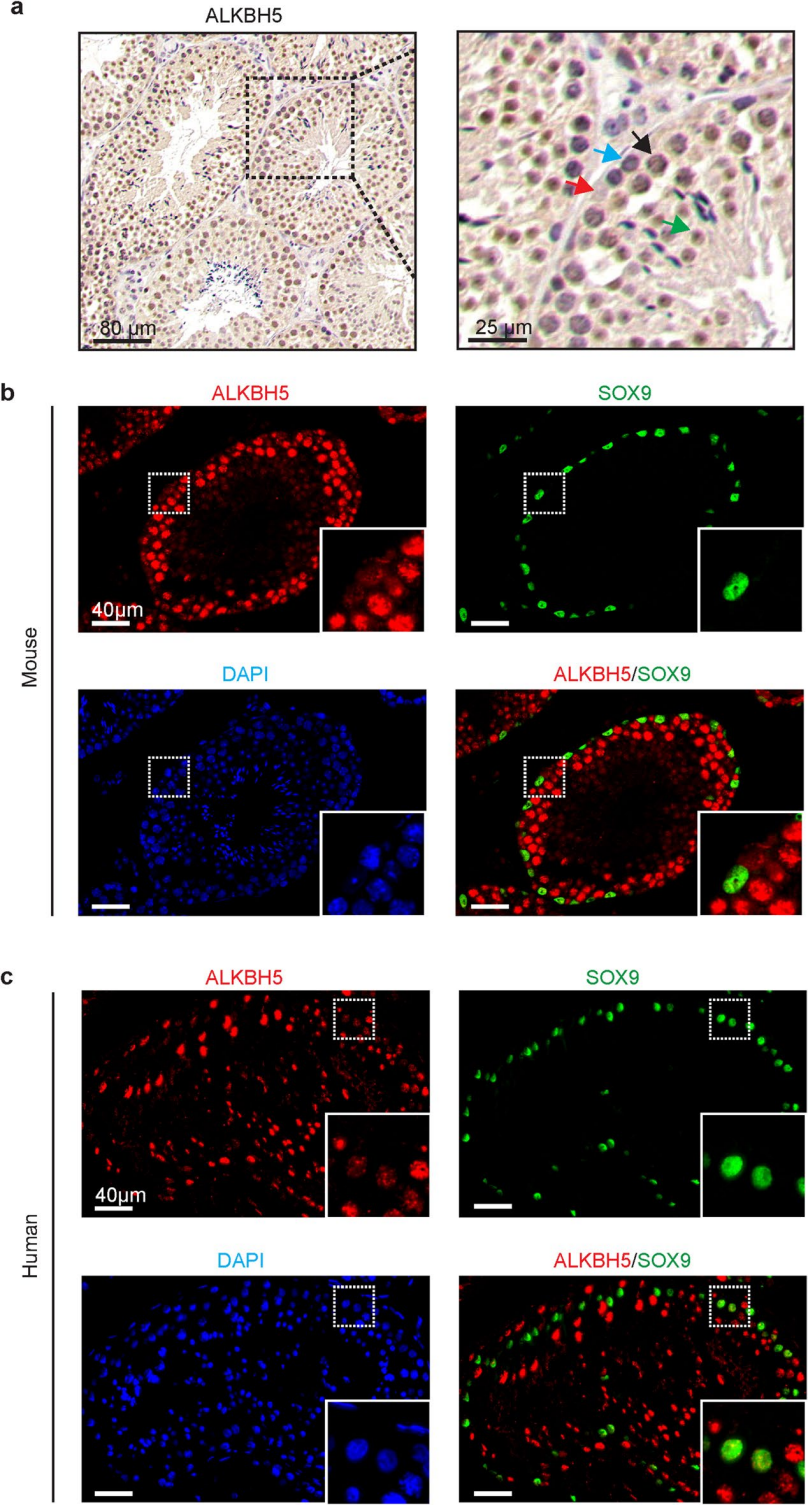
ALKBH5 expression in human and mouse testes. **a** Immunohistochemical analysis showing ALKBH5 expression in the testis of wild-type mice (*n* = 3). Red arrow, Sertoli cell; blue arrow, spermatogonia; black arrow, spermatocytes; green arrow, spermatids; **b**, **c** Immunofluorescence analysis showing ALKBH5 expression (SOX9, a marker of testicular Sertoli cells) in mouse (*n* = 3) (**b**) and human (*n* = 3) (**c**) testes with normal spermatogenesis

To understand the expression levels and distribution of ALKBH5 in adult testes, we first detected the expression of ALKBH5 in adult mouse testes. Similar to previous studies [1], ALKBH5 was significantly overexpressed in spermatocytes and spermatids. Meanwhile, it was also expressed, although at a relatively lower level, in other types of spermatogenic cells and somatic cells (Fig. 1a). Subsequently, we labeled Sertoli cells with SOX9 and confirmed the expression of ALKBH5 in Sertoli cells (Fig. 1b). Intriguingly, we found that, similar to its expression in the mouse testis, ALKBH5 was also expressed in Sertoli cells in human testes (Fig. 1c).

**Fig. 2:**
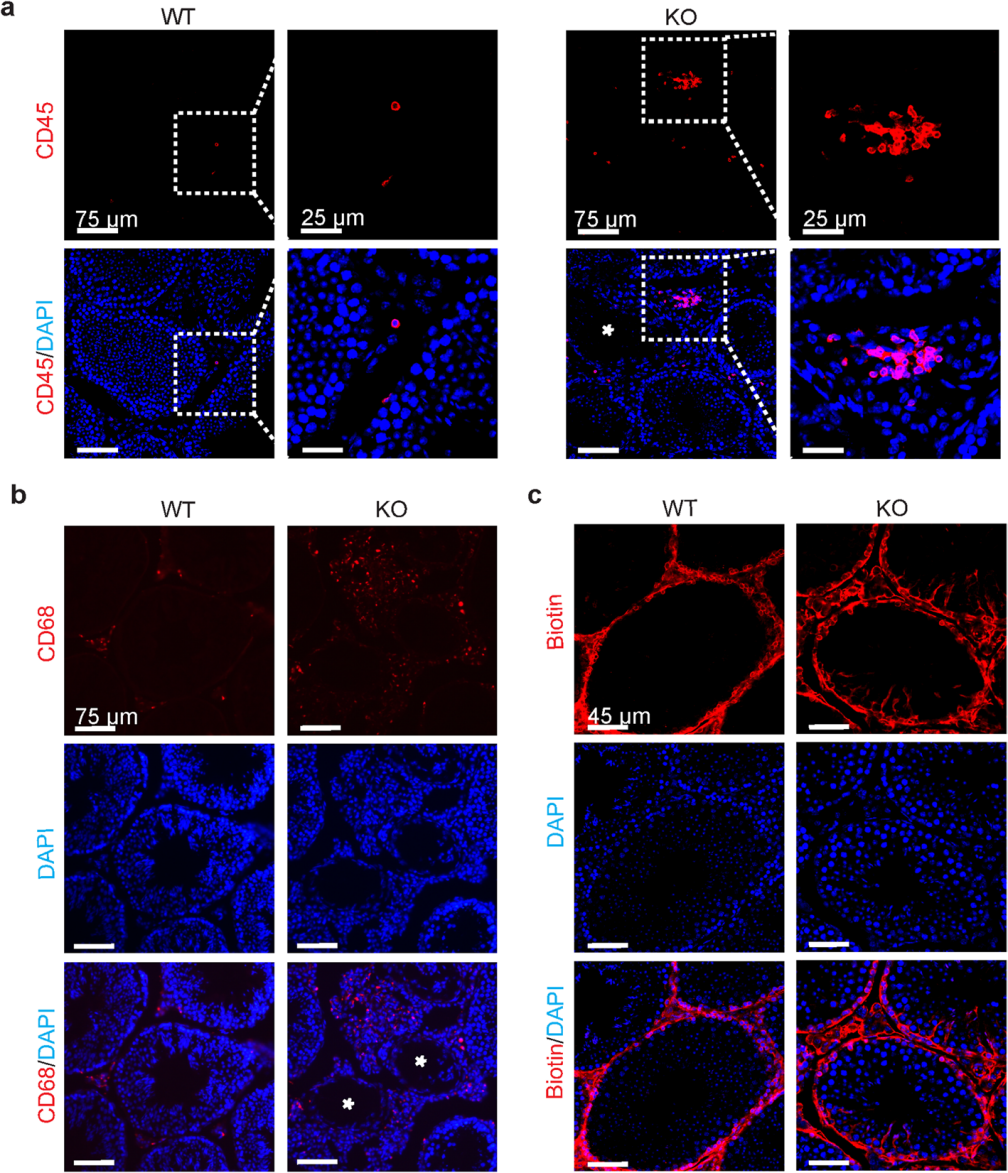
Comparison of the integrity of the blood–testis barrier between WT and *Alkbh5*-deficient mice. **a**, **b** Immunofluorescence analysis showing CD45^+^ (**a**) and CD68^+^ (**b**) cells infiltrated into the stroma in the testis of wild-type (WT) (*n* = 3) and *Alkbh5*-deficient (KO) (*n* = 3) mice, respectively (asterisk indicates morphologically damaged seminiferous tubule). **c** BTB assay detecting the integrity of the blood–testis barrier in WT (*n* = 3) and *Alkbh5*-KO mice (*n* = 3)

The lumen of the seminiferous tubule is an immune-privileged environment dependent on a tight BTB [7]. In the testis stroma of wild-type (WT) mice, we observed that very few CD45^+^ and some CD68^+^ cells appeared. In *Alkbh5*-knockout mice (*Alkbh5*-KO), around the seminiferous tubules, the extent of their infiltration into the stroma with normal morphology was similar to that in the wild-type mice (Fig. 2a, b). However, the numbers of CD45^+^ and CD68^+^ cells were increased significantly in the stroma near the morphologically damaged seminiferous tubules (Fig. 2a, b). Next, we performed a BTB assay to determine whether the aggregation of immune cells in the stroma may be caused by serious seminiferous tubule damage via destruction of the BTB. As shown in Fig. 2c, the biotin level in the testis of WT mice was limited to the stroma only, while in the testis of *Alkbh5*-KO mice, biotin was distributed not only in the stroma but also in the seminiferous tubules, regardless of the morphological integrity of the seminiferous tubule.

**Fig. 3:**
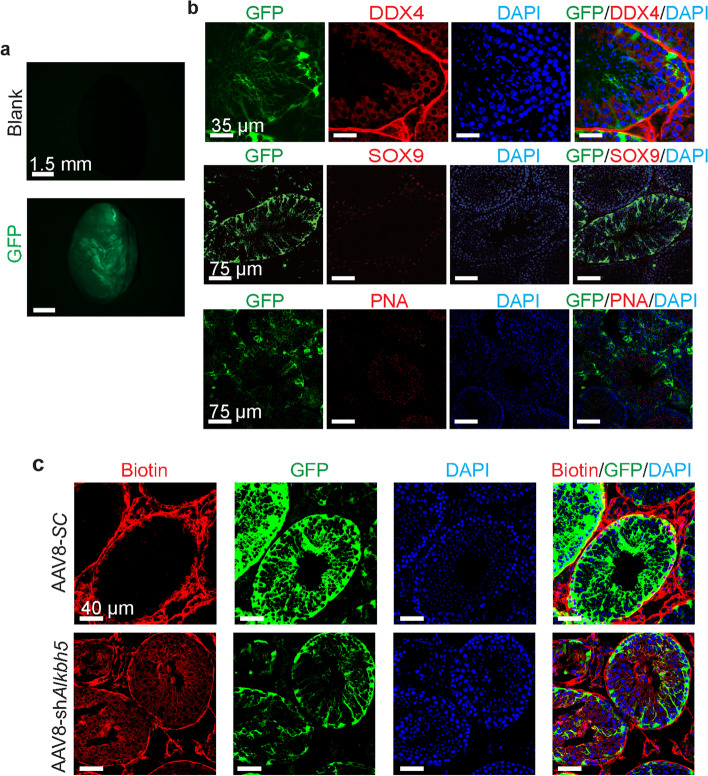
Destroyed integrity of the blood–testis barrier in mice with *Alkbh5* knockdown in Sertoli cells mediated by AAV8 infection. **a** Images showing GFP fluorescence in the testes infected with AAV8-scramble-GFP. **b** Immunofluorescence analysis to confirm the specificity of AAV8 infection in Sertoli cells (*n* = 3). DDX4, marker for germ cells but nonspermatid; SOX9, Sertoli cell marker; PNA, spermatid marker. **c** BTB assay detecting the integrity of the blood–testis barrier affected by AAV-mediated *Alkbh5* knockdown specifically in mouse Sertoli cells

To further clarify that ALKBH5 in Sertoli cells is related to BTB integrity, we constructed a mouse model with *Alkbh5* being knocked down specifically in Sertoli cells by microinjecting AAV8-sh*Alkbh5*-GFP particles into seminiferous tubules (Fig. 3a). One month later, we detected the expression of GFP in Sertoli cells but not in spermatogenic cells or outside of seminiferous tubules (Fig. 3b), indicative of the specificity of AAV8 infection in Sertoli cells. Next, the BTB assay showed that BTB integrity was damaged in the seminiferous tubules infected with AAV8-sh*Alkbh5*-GFP particles but not those infected with AAV8-scramble-GFP (Fig. 3c). These results suggest that ALKBH5 in Sertoli cells is involved in the regulation of BTB integrity.

### ALKBH5 regulates m^6^A levels on ***Cdh2*** mRNA and its translation

**Fig. 4:**
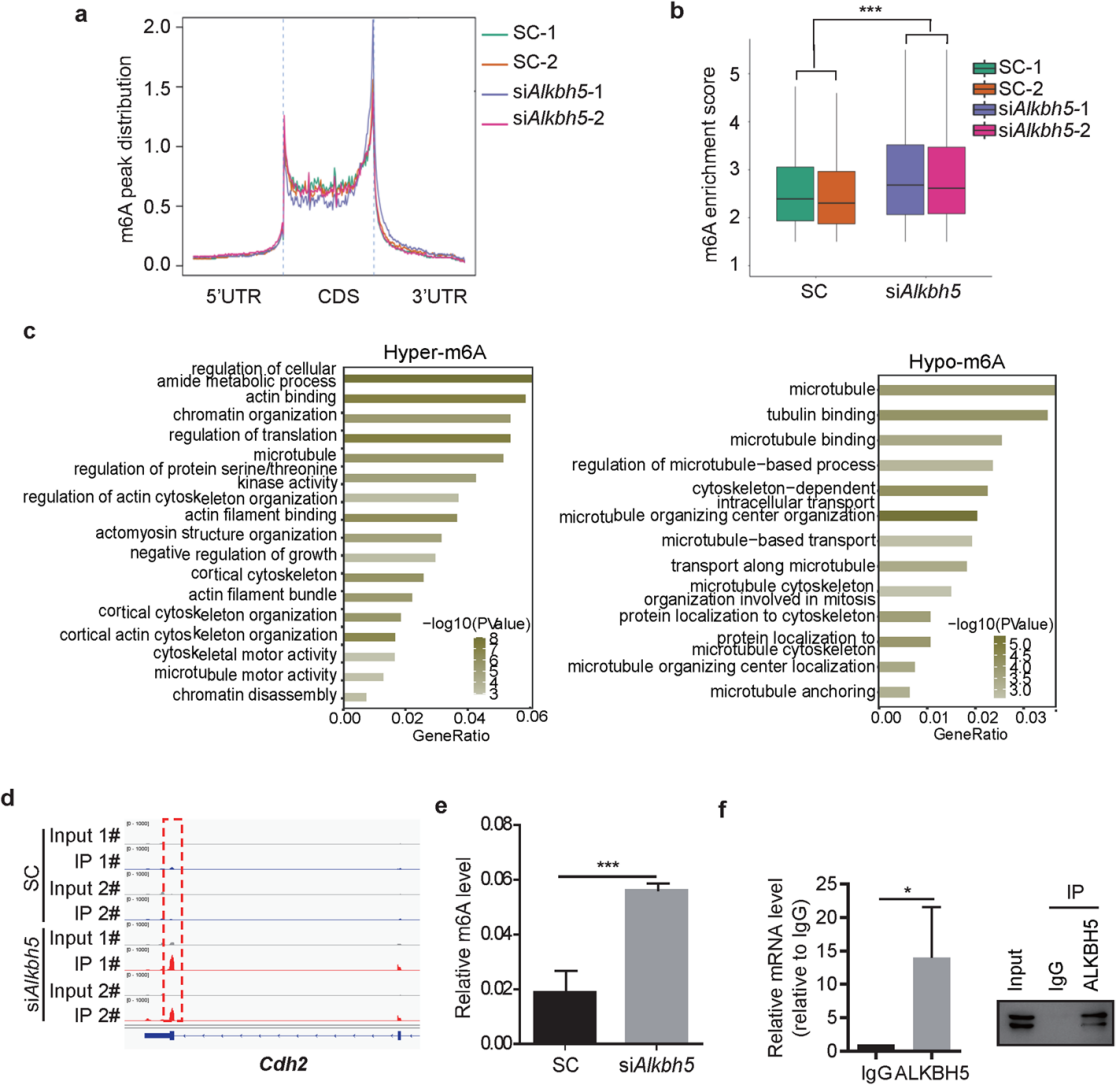
Identification of *Cdh2* mRNA as a substrate of ALKBH5 in the TM4 cell line. **a** Bioinformatic analysis for distribution of m^6^A peaks along the whole transcript. **b** Bioinformatic analysis for changes in the m^6^A enrichment score. ** c** Biological process analysis in Gene Ontology of the genes encoded by the hypermethylated or hypomethylated RNAs. **d** The intergrative Genomics Viewer (IGV) plots showing the m^6^A peak (marked by red dashed box) of *Cdh2* mRNA in TM4 cells transfected with scramble siRNA (SC) or *Alkbh5*-targeting siRNAs (si*Alkbh5*). **e** Results of m^6^A-IP qPCR showing the relative m^6^A level of *Cdh2* mRNA. **f** Results of RIP–qPCR detecting the interaction between ALKBH5 protein and *Cdh2* mRNA. Western blot analysis of the immunoprecipitated proteins is shown in the right panel. *P < 0.05

To investigate the intrinsic mechanism, we knocked down *Alkbh5* in mouse Sertoli cells TM4 and performed m^6^A-seq (Fig. 4a). Motif-searching analysis with all m^6^A peaks showed that all samples had conserved consensus motifs as reported in previous studies (Additional file 1: Fig. S1a) [12, 20]. After silencing *Alkbh5*, we observed an increase in the overall m^6^A level and identified 1416 hypermethylated RNAs and 615 hypomethylated RNAs (Fig. 4b, Additional file 2). Biological process analysis in Gene Ontology of the genes with upregulated and downregulated m^6^A levels showed some gene sets associated with the cytoskeleton for forming the BTB (Fig. 4c). We screened for BTB-related molecules reported previously in a review, in which main proteins related to tight junction, ectoplasmic specialization, desmosome, and gap junction at the blood–testis barrier were summarized [9]. As a result, we found that silencing *Alkbh5* caused a significant increase in the m^6^A level of *Cdh2* mRNA, which encodes an important structural protein, N-cadherin, in the BTB (Fig. 4d). The m^6^A IP-qPCR results confirmed that the m^6^A level on *Cdh2* mRNA increased after silencing *Alkbh5* (Fig. 4e). Furthermore, we observed that *Cdh2* mRNA was bound to ALKBH5 protein (Fig. 4f), suggesting that *Cdh2* mRNA is a demethylation substrate of ALKBH5 in Sertoli cells.

**Fig. 5:**
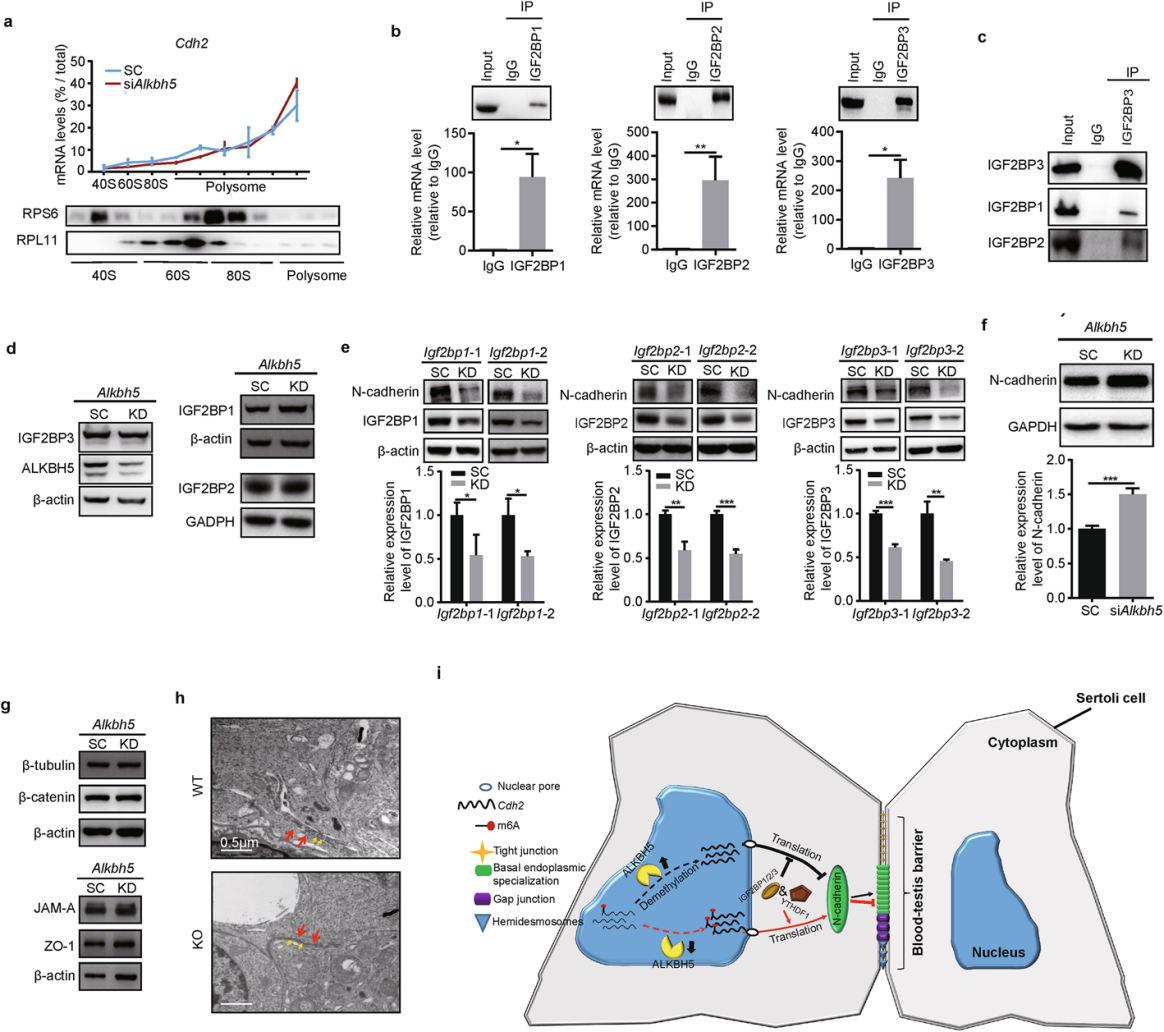
ALKBH5 regulates *Cdh2* mRNA translation via the IGF2BP1/2/3 complex and basal endoplasmic specialization via N-cadherin. **a** Polysome profiling analysis for detecting the translation efficiency of *Cdh2* mRNA in TM4 cells with and without *Alkbh5* knockdown. Western blot analysis was performed by using RPS6 and RPL11 to detect the efficiency of polysome fractionation. **b** RIP–qPCR to detect the interaction between IGF2BP1, IGF2BP2, or IGF2BP3 proteins and *Cdh2* mRNA. **c** Coimmunoprecipitation to detect the interaction between IGF2BP1, IGF2BP2, and IGF2BP3 proteins. **d** Western blot analysis to detect the expression of IGF2BP1, IGF2BP2, and IGF2BP3 after silencing *Alkbh5* in TM4 cells. β-Actin and GAPDH were used as internal controls. **e** Western blot analysis to detect the expression of N-cadherin after knocking down *Igf2bp1*, *Igf2bp2*, or *Igf2bp3*. β-Actin was used as an internal control. **f** Western blot analysis showing the expression of N-cadherin before and after knocking down *Alkbh5*. GAPDH was used as an internal control. **g** Some key BTB-related molecules detected by western blot analysis. β-Actin was used as an internal control. **h** Images showing the ultrastructure of basal endoplasmic specialization (marked by red arrow) in the testis of WT or *Alkbh5*-KO mice as observed under transmission electron microscopy. Yellow arrow indicates actin bundles. **i** Schematic diagram elucidating the mechanism of ALKBH5 in regulating the integrity of the blood–testis barrier. Under physiological conditions with normal ALKBH5 expression, *Cdh2* mRNA is maintained at a relatively low m^6^A level and is kept away from the IGF2BP1/2/3 complex and YTHDF1-mediated translation of *Cdh2*. Thus, N-cadherin is expressed at a reasonable level to maintain the stability of basal endoplasmic specialization and integrity of the blood–testis barrier. However, insufficient ALKBH5 protein expression leads to hypermethylation of *Cdh2* mRNA and further facilitates its translation under the action of the IGF2BP1/2/3 complex and YTHDF1. Consequently, overexpression of N-cadherin disrupts the stability of basal endoplasmic specialization and integrity of the blood–testis barrier. *P < 0.05, **P < 0.01, ***P < 0.001

Next, we aimed to elucidate the molecular mechanism underlying ALKBH5-mediated regulation of *Cdh2* mRNA. We knocked down *Alkbh5* but observed no change in the expression of *Cdh2* mRNA (Additional file 1: Fig. S1b), which was consistent with the RNA-seq data of *Alkbh5*-knockdown cells (Additional file 3). Next, we analyzed the changes in all alternative splicing events induced by *Alkbh5* knockdown. Although multiple RNAs exhibited significant changes in alternative splicing events (Additional file 1: Fig. S1c), we failed to detect any significant change with *Cdh2* mRNA after silencing *Alkbh5* (Additional file 1: Fig. S1d). Notably, a polysome profiling assay showed that the *Cdh2* mRNA translation efficacy after silencing *Alkbh5* had an upward trend, although there was no statistical difference (Fig. 5a). Therefore, we detected the relationship between several translation-related m^6^A-binding proteins and *Cdh2* mRNA. As shown in Fig. 5b, we detected a strong interaction between *Cdh2* mRNA and IGF2BP1, IGF2BP2, and IGF2BP3 proteins, respectively. Interestingly, coimmunoprecipitation showed that IGF2BP3 interacted with IGF2BP1 and IGF2BP2 (Fig. 5c). Although ALKBH5 did not affect the protein expression of IGF2BP1, IGF2BP2, or IGF2BP3 (Fig. 5d), silencing either *Igf2bp1*, *Igf2bp2* or *Igf2bp3* led to a significant decrease in the expression of N-cadherin (Fig. 5e). The above results indicate that ALKBH5 regulates the m^6^A level of *Cdh2* mRNA, which further affects its translation with the aid of IGF2BP1/2/3 complexes. Additionally, we also found that YTHDF1, another translation-related m^6^A binding protein, interacted with *Cdh2* mRNA (Additional file 1: Fig. S1e), and silencing *Ythdf1* also led to decreased N-cadherin expression (Additional file 1: Fig. S1f), but silencing *Alkbh5* did not change YTHDF1 expression (Additional file 1: Fig. S1g). We speculated that YTHDF1 also mediated N-cadherin translation in our study. In summary, ALKBH5 regulates m^6^A levels on *Cdh2* mRNA and its translation via the IGF2BP1/2/3 complex and YTHDF1.

### ALKBH5 regulates basal endoplasmic specialization via N-cadherin

Finally, we found that N-cadherin expression, which is encoded by *Cdh2*, was indeed upregulated after silencing *Alkbh5* (Fig. 5f). Additionally, we also observed that N-cadherin was mainly distributed at the basal compartment of seminiferous tubules in normal testes, and its distribution was more diffuse at seminiferous tubules after *Alkbh5* knockout (Additional file 1: Fig. S1h). It is known that the BTB is mainly composed of tight junctions, basal endoplasmic specializations, gap junctions, and hemidesmosomes, while N-cadherin is an important structural molecule of basal endoplasmic specialization [21]. Although *Alkbh5* knockdown did not induce any change in other key BTB-related molecules (Fig. 5g), we indeed found that, in contrast to the tidy actin bundles in *Alkbh5*-WT mice, the actin bundles of basal endoplasmic specializations in the testis of *Alkbh5*-KO mice were severely disordered (Fig. 5h). In summary, our study suggested that ALKBH5 in mouse testicular Sertoli cells regulates *Cdh2* mRNA translation to maintain blood–testis barrier integrity (Fig. 5i).

## Discussion

To date, the role of ALKBH5 in spermatogenic cells and Leydig cells has been studied, but there is a lack of research on the role of ALKBH5 in testicular Sertoli cells. In this study, the BTB assay showed that *Alkbh5* depletion in Sertoli cells led to the destruction of BTB integrity. Further studies found that ALKBH5 regulated the m^6^A level of *Cdh2* mRNA and promoted its translation with the aid of the IG2BP1/2/3 complex and YTHDF1. Finally, ALKBH5 participates in the assembly of basal endoplasmic specialization through *Cdh2*-coding N-cadherin.

The lumen of the seminiferous tubule is an immune-privileged environment [22]. BTB can prevent immune cells from entering the lumen and disrupt spermatogenic cells [22]. Under normal conditions, there are a small number of immune cells in testicular stroma [22]. Upon BTB damage, a large number of immune cells infiltrate the testicular stroma owing to lumen exposure. In this study, we found that there were a large number of immune cells in the stroma around the failed seminiferous tubules of *Alkbh5*-KO mice, hinting at BTB destruction. The BTB assay confirmed that *Alkbh5* knockdown or knockout led to damage to the BTB. Additionally, it is known that the cytoskeleton, including actin and microtubules, is an important infrastructure for forming the BTB [23] and that imbalanced cytoskeletal systems destroy the BTB. We found that genes with m^6^A upregulation after *Alkbh5* knockdown were involved in the cytoskeletal function of actin or microtubules. *Cdh2-*encoding N-cadherin is the main structural molecule of basal endoplasmic specialization. In this study, we found that *Cdh2* mRNA translation was upregulated by silencing *Alkbh5* and that basal endoplasmic specialization in the testes of KO mice was seriously disturbed. Therefore, we deduced that ALKBH5 participates in BTB formation via basal endoplasmic specialization.

ALKBH5 is expressed in mouse spermatogenic cells, Leydig cells, and Sertoli cells [24]. In this study, we confirmed that ALKBH5 was expressed in mouse testicular Sertoli cells. Meanwhile, we also determined that the expression pattern of ALKBH5 in human testis was similar to that in mouse testis. It has been reported that ALKBH5 in the testis regulates RNA metabolism. In spermatogenic cells, ALKBH5 is involved mainly in regulating the alternative splicing of mRNA and circular RNA [4, 5]. In Leydig cells, ALKBH5 also participates in regulating mRNA stability [8]. In this study, we found that, in Sertoli cells, ALKBH5 promoted *Cdh2* translation. N-cadherin is known to be an important protein for BTB via basal endoplasmic specialization. Cumulative studies have shown that the downregulation of N-cadherin is one of the important reasons for the destruction of BTB integrity [25–28]. In our study, we found that knocking down *Alkbh5* increased N-cadherin expression and that basal endoplasmic specialization was disrupted in *Alkbh5*-KO mice. It is known that gene expression in cells is under precise regulation, and the imbalance would cause functional disruptions. Therefore, we thought that ALKBH5 can enhance BTB integrity by inhibiting the overexpression of N-cadherin. Finally, we found that IGF2BP1, IGF2BP2, and IGF2BP3 bound to *Cdh2* mRNA and regulated its translation. It has been shown that IGF2BP1, IGF2BP2, and IGF2BP3 promote the translation of target genes [29, 30]. In addition, some studies have shown that there is a synergistic effect between different m^6^A binding proteins to regulate RNA metabolism [30, 31]. In this study, we found an interaction between IGF2BP1, IGF2BP2, and IGF2BP3 proteins. Therefore, it is thought that IGF2BP1, IGF2BP2, and IGF2BP3 cooperate to promote *Cdh2* mRNA translation. However, in this study, we found that YTHDF1 also bound to *Cdh2* mRNA, and knockdown of *Ythdf1* also led to decreased N-cadherin expression. Cumulative studies have shown that YTHDF1 is responsible for the translation of target genes via m^6^A [32–35]. Therefore, we speculated that both the IGF2BP1/2/3 complex and YTHDF1 in Sertoli cells are involved in the regulation of *Cdh2* mRNA translation. However, more experiments are needed to clarify the mechanism by which the above m^6^A regulators promote translation.

Normal spermatogenesis depends on an intact BTB to prevent toxic substances and immune cells from invading the seminiferous tubules. Cumulative studies have shown that some adverse factors, such as new coronavirus [36], Marburg virus [37], chemotherapeutic drug busulfan [38], and heavy metal ion cadmium [39], can damage the BTB, resulting in spermatogenesis disorder and even male infertility. In addition, some studies have shown that some substances, such as manganese [40], nicotine and its metabolites in tobacco smoke [41], and metal oxide nanoparticles [42], can directly pass through the BTB and cause spermatogenesis disorder. Therefore, maintaining BTB integrity is an important basis for normal spermatogenesis. Fortunately, some drugs, such as *Lycium barbarum* polysaccharide [43] and traditional Chinese medicine [44, 45], have been found to maintain BTB integrity and promote spermatogenesis. In this study, we confirmed that ALKBH5 in Sertoli cells is involved in regulating BTB integrity. Our previous study found that *ALKBH5* mRNA level was downregulated in patients with idiopathic azoospermia [46]. Further analysis also showed that ALKBH5 was dysregulated in testicular Sertoli cells in patients with idiopathic azoospermia (unpublished data). All these studies demonstrate that ALKBH5 plays an important role in testicular spermatogenesis, and the aberrant ALKBH5 expression in Sertoli cells is an important molecular pathological change that cannot be ignored for male infertility.

In conclusion, in this study, we found that ALKBH5 regulates the m^6^A level of *Cdh2* mRNA in mouse testicular Sertoli cells and that *Cdh2* mRNA translation is mediated by IGF2BP1/2/3 complexes and YTHDF1. Ultimately, we discovered that ALKBH5 participates in the regulation of basal endoplasmic specializations to regulate BTB integrity via N-cadherin.

### Supplementary Information

**Additional file 1.** Supplementary studies on the mechanisms of ALKBH5 on BTB integrity and list of antibodies used in this study.

**Additional file 2.** Bioinformatic analysis results of m6A-seq data after sliencing *Alkbh5* in TM4 cell line.

**Additional file 3.** List of differentially expressed genes after sliencing *Alkbh5* in TM4 cell line.

## Data Availability

All data needed to evaluate the conclusions in the paper are present in the paper or the Supplementary Materials. The raw m^6^A-seq data presented in this study have been deposited in the Genome Sequence Archive in BIG Data Center, Beijing Institute of Genomics (BIG), Chinese Academy of Sciences, under accession number CRA006717 that is accessible at https://bigd.big.ac.cn. Materials described in the study are available on request from the corresponding author.

## Abbreviations

AS: Alternative splicing
BTB: Blood–testis barrier
BTB assay: Blood–testis barrier permeability assay
GO: Gene Ontology
IF: Immunofluorescence
IHC: Immunohistochemical staining
IP: Immunoprecipitation
KO: Knockout
m^6^A-seq: m^6^A-immunoprecipitation sequencing
OCT: Optimal cutting temperature compound
RIP: RNA immunoprecipitation
siRNA: Small interfering RNA
WB: Western blotting
WT: Wild type
