# Correction: ALKBH5 in mouse testicular Sertoli cells regulates *Cdh2* mRNA translation to maintain blood–testis barrier integrity

**DOI:** 10.1186/s11658-024-00534-4

**Published:** 2024-01-18

**Authors:** Zhonglin Cai, Yao Zhang, Lin Yang, Chunhui Ma, Yi Fei, Jing Ding, Wei Song, Wei-Min Tong, Yamei Niu, Hongjun Li

**Affiliations:** 1 Department of Urology, Peking Union Medical College Hospital, Chinese Academy of Medical Sciences & Peking Union Medical College, Beijing, China; 2 Department of Pathology, Institute of Basic Medical Sciences Chinese Academy of Medical Sciences, School of Basic Medicine Peking Union Medical College, Beijing, China; 3 Department of Urology, Shanghai Ninth People’s Hospital, Shanghai Jiaotong University School of Medicine, Shanghai, China; 4 Department of Biochemistry and Molecular Biology, State Key Laboratory of Medical Molecular Biology, Institute of Basic Medical Sciences Chinese Academy of Medical Sciences, School of Basic Medicine Peking Union Medical College, Beijing, China; 5 Molecular Pathology Research Center, Chinese Academy of Medical Sciences and Peking Union Medical College, Beijing, China

**Correction** **:** **Cellular & Molecular Biology Letters (2022) 27:101** 10.1186/s11658-022-00404-x

Following publication of the original article [1], the authors corrected the Funding section.

The incorrect Funding is:

- This work is supported by the Grant from National Natural Science Foundation of China (81871152, 82171588), National Key R&D Program of China (2019YFA080703), and Chinese Academy of Medical Sciences (CAMS) Initiative for Innovative Medicine (2021-I2M-1-002).

The correct Funding is:

- This work is supported by the Grant from National Natural Science Foundation of China (81871152, 82171588), National Key R&D Program of China (2019YFA0801703), and Chinese Academy of Medical Sciences (CAMS) Initiative for Innovative Medicine (2021-I2M-1-002).
