# Supplementary material for: ALKBH5 in mouse testicular Sertoli cells regulates Cdh2 mRNA translation to maintain blood–testis barrier integrity
Source: Cell Mol Biol Lett. 2022 Nov 22;27:101. doi: 10.1186/s11658-022-00404-x (PMC9682758; doi:10.1186/s11658-022-00404-x)
Supplement: Supplementary file 1 — Additional file 1. Supplementary studies on the mechanisms of ALKBH5 on BTB integrity and list of antibodies used in this study. [file 11658_2022_404_MOESM1_ESM.docx]

**Supplementary Information**

ALKBH5 in mouse testicular Sertoli cells regulates *Cdh2* mRNA translation to maintain blood-testis barrier integrity

^1^Department of Urology, Peking Union Medical College Hospital, Peking Union Medical College, Chinese Academy of Medical Sciences, Beijing, China

^2^Department of Pathology, Institute of Basic Medical Sciences, Chinese Academy of Medical Science and School of Basic Medicine, Peking Union Medical College, Beijing, China.

^3^Department of Urology, Shanghai Ninth People's Hospital, Shanghai Jiaotong University School of Medicine, Shanghai, China.

^4^Department of Biochemistry and Molecular Biology, State Key Laboratory of Medical Molecular Biology, Institute of Basic Medical Sciences, Chinese Academy of Medical Sciences and School of Basic Medicine, Peking Union Medical College, Beijing 100005, China.

^5^Molecular Pathology Research Center, Chinese Academy of Medical Sciences and Peking Union Medical College, Beijing, 100005, China

**Running title:** ALKBH5 and blood-testis barrier

**Key words:** RNA N6-methyladenosine, *Alkbh5*, blood-testis barrier, *Cdh2*, basal endoplasmic specialization

***Corresponding author 1:** Hongjun Li, Tel: +86-10-69156034. E-mail: lihongjun@pumch.cn

***Corresponding author 2:** Yamei Niu, Tel: +86-10-69156945; E-mail: niuym@ibms.pumc.edu.cn

***Corresponding author 3:** Wei-min Tong, Tel: +86-10-69156945; E-mail: wmtong@ibms.pumc.edu.cn

**Disclosure of potential conflicts of interest:** None

**Fund:** This work is supported by the grant from National Natural Science Foundation of China (81871152, 82171588), National Key R&D Program of China (2019YFA080703), and Chinese Academy of Medical Sciences (CAMS) Initiative for Innovative Medicine (2021-I2M-1-002).

**This PDF file includes:**

Figure S1

Table S1

**Figure S1**


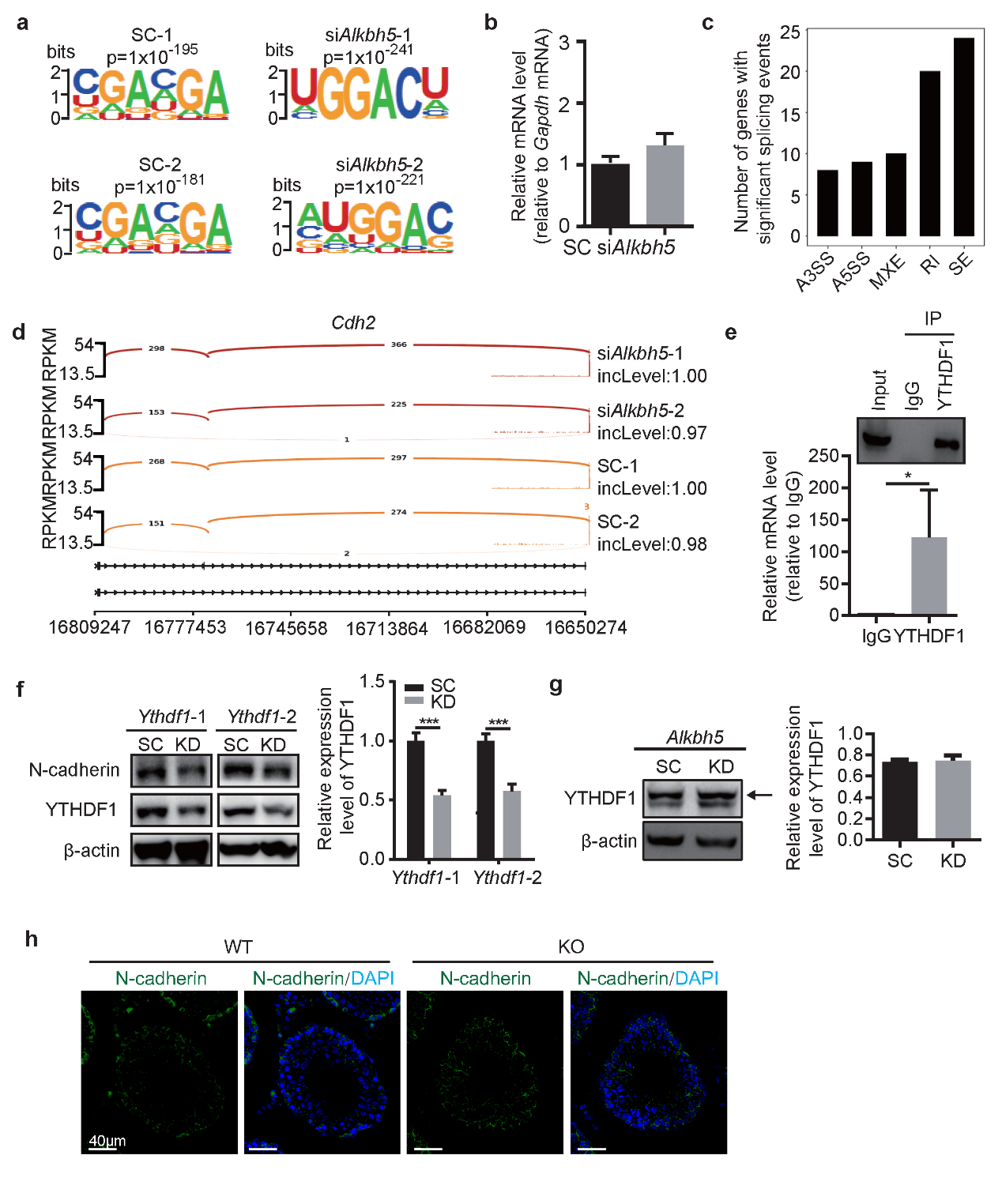


**Figure S1** Supplementary studies on the mechanisms of ALKBH5 on BTB integrity. **a** Motif analysis for m^6^A consensus motifs; **b** RT–qPCR to detect the change in *Cdh2* mRNA expression; **c** Alternative splicing analysis including alternative 5' splice site (A5SS), alternative 3' splice site (A3SS), skipped exon (SE), mutually exclusive exons (MXE) and retained intron (RI) for all genes; **d** Alternative splicing analysis for *Cdh2* mRNA; **e** RIP-qPCR to detect the interaction between YTHDF1 protein and *Cdh2* mRNA; **f** Western blot analysis to detect the expression of N-cadherin after knocking down *Ythdf1*. β-actin was used as an internal control; **g** Western blot analysis showing YTHDF1 expression after silencing *Alkbh5*. β-actin was used as an internal control; **h** Immunofluorescent analysis showing N-cadherin expression distribution in the WT (n=3) and *Alkbh5*-KO mice (n=3). * P<0.05, *** P<0.001.

| **Table S1 List of antibodies and their applications used in this study.** | | | |
| --- | --- | --- | --- |
| **Antibody** | **Source** | **Code** | **Application** |
| Anti-ALKBH5 | Sigma | HPA007196 | WB, IHC, IF |
| Anti-β-actin | Santa Cruz | sc-47778 | WB |
| Anti-GAPDH | Cell Signaling Technology | 2118 | WB |
| Anti-m^6^A | Synaptic Systems | 202003 | m^6^A-IP |
| Anti-Goat IgG (H+L)-HRP conjugated | XI YA Biology | FZ-4211 | WB |
| Anti-Mouse IgG (H+L)-HRP conjugated | XI YA Biology | FZ-4202 | WB |
| Anti-N-cadherin | Cell Signaling Technology | 13116 | WB |
| Anti-ALKBH5 | MBL | RN122PW | RIP |
| Anti-SOX9 | abcam | ab185966 | IF |
| Anti-IGF2BP1 | Cell Signaling Technology | 8482 | WB, RIP |
| Anti-IGF2BP2 | Proteintech | 11601-1-AP | WB, RIP |
| Anti-YTHDF1 | Proteintech | 17479-1-AP | WB, RIP |
| Anti-IGF2BP3 | Abcam | ab177477 | WB, Co-IP, RIP |
| Anti-RPL11 | Cell Signaling Technology | 18163 | WB |
| Anti-RPS6 | Abcam | ab225676 | WB |
| Anti-CD45 | Abcam | ab10558 | IF |
| Anti-CD68 | Cell Signaling Technology | 97778 | IF |
| Anti-DDX4 | Abcam | ab13840 | IF |
| Anti-PNA-FITC | Sigma | L7381 | IF |
| Anti-β-tubulin | Abcam | ab6046 | WB |
| Anti-β-catenin | Thermo | 71-2700 | WB |
| Anti-JAM-A | Thermo | 36-1700 | WB |
| Anti-ZO-1 | invitrogen | 61-7300 | WB |
| Abbreviation: WB, western blot; IHC, immunohistochemical staining; IF, immunofluorescence; Co-IP, co-immunoprecipitation; RIP: RNA immunoprecipitation | | | |
